# A Short Reference Differential Chaos Shift Keying Cooperative Communication System Based on Modified Code Index Modulation

**DOI:** 10.3390/e27060562

**Published:** 2025-05-26

**Authors:** Bin Yu, Hua Yang, Yaqiong Jia, Hao Liao, Xin Li

**Affiliations:** 1 School of Electrical Information Engineering, Hunan Institute of Technology, Hengyang 421002, China; jyqhugong@hnit.edu.cn (Y.J.); 2003001368@hnit.edu.cn (H.L.); 2021001010@hnit.edu.cn (X.L.); 2 School of Computer Science, Nanjing University of Posts and Telecommunications, Nanjing 210023, China; 3 Micro and Nano Optoelectronic Devices and Integrated Technology Key Laboratory of Universities in Hunan Province, Hengyang 421002, China; 4 School of Electronic and Optical Engineering, Nanjing University of Posts and Telecommunications, Nanjing 210023, China; yangh@njupt.edu.cn

**Keywords:** chaotic communication, cooperative communication, short reference differential chaos shift keying (SR-DCSK), code index modulation

## Abstract

In this paper, a new short reference differential chaos shift keying (SR-DCSK) cooperative communication system based on modified code index modulation, referred to as the MCIM-SR-DCSK-CC system, is proposed. In the proposed MCIM-SR-DCSK-CC system, the information bits are transmitted to both the relay and the destination in the first time slot. On the other hand, in the second time slot, the relay not only forwards the information bits but also sends new information bits to the destination. Specifically, the relay adopts the modified code index modulation to transmit more information bits to the destination. The theoretical bit error rate (BER) expressions of the proposed MCIM-SR-DCSK-CC system are obtained over additive white Gaussian noise (AWGN) and the multipath Rayleigh fading channels. It is demonstrated that the simulation results of BER performance match the theoretical results. The energy efficiency (EE), the data rate, and transmission throughput are carefully analyzed. The performance of the proposed system is better than its competitors’.

## 1. Introduction

According to the characteristics of the chaotic signals, such as initial value sensitivity, noise-like characteristics, and wide spectrum, the chaotic signals are widely adopted in communication systems [1,2,3,4]. Among many chaotic modulation schemes, differential chaos shift keying (DCSK) is the leading non-coherent option [5,6], which has been extensively employed in various short-range wireless communication scenarios, like wireless sensor networks (WSNs) and wireless body area networks (WBANs) [7,8]. However, the traditional DCSK system allocates each data frame into two time slots, one for reference-chaotic signals and one for information-bearing signals. This allocation leads to low data rates, low spectral efficiency, and energy inefficiency. Hence, several viable methods have been suggested to overcome the mentioned limitations, such as short reference DCSK (SR-DCSK) [9], multi-carrier DCSK (MC-DCSK) [10], noise reduction DCSK (NR-DCSK) [11], high-efficiency DCSK (HE-DCSK) [12], and so on.

Recently, index modulation (IM) has been developed as a means to enhance the data rate and energy efficiency of chaos-based communication systems. In the IM technique, a variety of transmission resources, including spreading codes, time slots, subcarriers, and transmit antennas, can be used to transmit information [13,14]. To achieve higher data rates, the code index modulation (CIM) technique was first used in direct-spread spectrum-sequence communication systems [15,16]. The M-ary orthogonal multilevel differential chaos shift keying system with code index modulation (CIM-OM-MDCSK) is proposed for high data rate and high spectral efficiency [17]. In the CIM-OM-MDCSK system, the M-ary information signals consist of modulated bits, chaotic signals, and their Hilbert transforms, and the selected Walsh codes with specific indices are used to modulate the multiple information-bearing signals. The modified code index modulation scheme based on multi-carrier M-ary DCSK (MC-MCIM-MDCSK) is proposed for high energy efficiency (EE), better spectral efficiency (SE), and high data rate [18]. The carrier index (CI) scheme that carries additional information bits by activating different subcarriers was proposed in [19,20]. Then some modified carrier index differential chaos shift keying (CI-DCSK) systems have been proposed to obtain higher data rates, better energy efficiency, and spectrum efficiency. The carrier index M-ary differential chaos shift keying system (CI-MDCSK) transmitted the modulated bits by the active carriers through M-ary DCSK modulation, which is based on the Hilbert transform [21]. The high data rate carrier index differential chaos shift keying (HDR CI-DCSK) is proposed to obtain higher data rates, better energy efficiency, and spectrum efficiency [22]. The multi-carrier DCSK with hybrid index modulation (HIM-MC-DCSK) system is proposed by combining the carrier-number-index technique and carrier-index technique with MC-DCSK modulation to substantially enhance transmission efficiency in wireless communication systems [23].

Spatial diversity has also received considerable attention as another promising technique to combat multipath fading [24,25]. The use of multiple antennas to transmit redundant signal information can significantly mitigate the impact of multipath propagation. The cooperative communication, which provides transmit diversity by placing a relay between the source and destination, has been proposed as an alternative approach to tackle multipath fading [26]. Moreover, it has been shown in [27,28,29] that integrating DCSK with cooperative communication (CC) can substantially enhance the reliability of wireless communication systems. In [30], the partial-sequence CC (PS-CC) scheme is proposed for improving data rate. In order to enhance the transmission throughput, the IM technique can be employed in cooperative communication [31]. A two-way multi-user half-duplex cooperative communication system based on the joint grouping subcarrier-permutation index modulation-DCSK (GSPIM-DCSK) system is proposed in [32]. In [33], the SR-DCSK cooperative communication system with a code index modulation (CIM-SR-DCSK-CC) system is proposed in order to increase the throughput without negatively impacting the BER performance. In order to maintain the advantages of the CIM-SR-DCSK-CC system and improve the data rate, we propose an SR-DCSK cooperative communication system based on modified code index modulation (MCIM), referred to as the MCIM-SR-DCSK-CC system in this paper. The main contributions of this paper are as follows:

In the proposed MCIM-SR-DCSK-CC system, the SR-DCSK scheme is employed to achieve the communication between the source and the destination, and the modified code index modulation scheme based on M-ary DCSK (MCIM-MDCSK) is adopted to realize the communication between the relay and the destination. On the other hand, in the proposed system, each transmission period is divided into two phases: the source transmits the polarity-modulated bits to the relay and the destination in the first phase; the relay transmits its own information bits and the polarity-modulated bits to the destination based on a given decode-and-forward (DF) cooperative protocol in the second phase.

The DF protocol is a widely used relaying strategy in wireless communication systems, particularly in cooperative communication scenarios. It is designed to improve the reliability and performance of communication links by leveraging intermediate relay nodes [33]. The DF protocol operates in two main phases: the transmission phase and the relay phase. In the transmission phase, the information signal is transmitted from the source to the relay. The source node S transmits the data signal to the relay node R. The relay node R receives the signal, which may be corrupted by noise and channel impairments. The relay node attempts to decode the received signal. If the signal is successfully decoded, the relay node prepares to forward the data to the destination node D. In the relay phase, the information signal is transmitted from relay to destination. After successfully decoding the signal, the relay node R re-encodes the data and retransmits them to the destination node D. The destination node D receives the signal from both the source node S and the relay node R. The destination node combines the received signals (e.g., using maximum ratio combining or selection combining) to improve the reliability of the received data. The destination node decodes the combined signal to retrieve the original data.

The theoretical BER formula of the proposed MCIM-SR-DCSK-CC system is obtained over the AWGN and multipath Rayleigh fading channels. The simulation results closely match the theoretical analysis results. The comparative analysis between the proposed system and the CIM-SR-DCSK-CC system is given to confirm the excellence of the proposed system.

The EE, data rate, and throughput of the proposed system are analyzed and compared with other DCSK cooperative communication systems. The proposed system has better EE, a higher date rate, and higher throughput than its competitor. The computational complexity and the memory requirement are also analyzed.

The rest of this paper is as follows: The system model of the proposed system is given in Section 2. The theoretical analysis of the BER performance, the EE, the data rate, the throughput, the computational complexity, and the memory requirement are shown in Section 3. Section 4 shows the simulation results and discussions. Section 5 gives the conclusion.

## 2. System Model

### 2.1. Proposed MCIM-SR-DCSK-CC System

**Figure 1:**
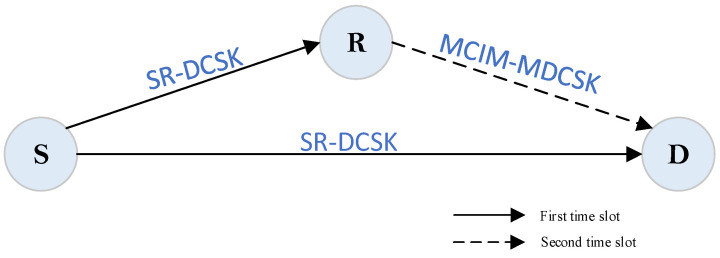
The proposed MCIM-SR-DCSK-CC model.

A half-duplex cooperative communication system is considered in this paper. The general system model is shown in Figure 1. The proposed system includes a source node S, a relay node R, and a destination node D. In the proposed system, the information bit streams include polarity-modulated bits, code index bits, and code-modulated bits. The polarity-modulated bits are transmitted by S. The code index bits and code-modulated bits are transmitted by R. Each transmission period is composed of two time slots. There are N groups of information bit streams.

### 2.2. S-R Link and S-D Link

**Figure 2:**
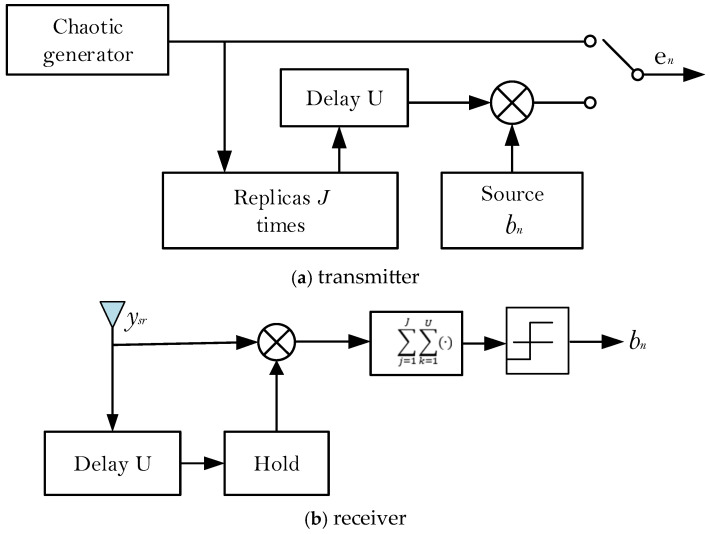
The system model of SR-DCSK.

**Figure 3:**
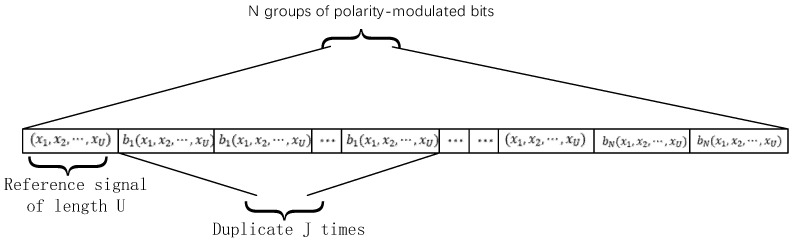
The frame structure of the transmitted signal at S.

The SR-DCSK modulation scheme is adopted at S. The transmitter and receiver system model of SR-DCSK are shown in Figure 2. The frame structure of the transmitted signal at S is shown in Figure 3. We take the nth group of information bit streams as an example. The nth polarity-modulated bit is first mapped to a polarity-modulated symbol $b_{n}$, which is carried by the SR-DCSK signal and simultaneously transmitted to R and D in the first time slot. R adopts the DF protocol to recover the polarity-modulated symbol and transmit the code index bits and code-modulated bits to D in the second time slot.

The chaotic signal $x=[x_{1},x_{2},\ldots x_{U}]$ is generated by a second-order Chebyshev polynomial function (i.e., $x_{k+1}=1-\varphi x_{k}^{2}$) with initial conditions $x_{1}=0.9058$ and the parameter φ=2. The length of the chaotic signal is U. The length of the information-bearing signal is β, where β=J·U. J is the number of replications. Thus, the transmitted signal of the nth polarity-modulated symbol $b_{n}$ can be denoted as

(1) $$e_{n}=[x,\underset{Duplicat\;J\;times}{\underbrace{b_{n}x,\ldots ,b_{n}x}}]$$

The signals received by R and D can be denoted as

(2) $$y_{sr,k}=\sum_{l=1}^{L_{sr}}\alpha_{sr,l}\cdot e_{sr,k-\tau_{sr,l}}+n_{sr,k}$$

(3) $$y_{sd,k}=\sum_{l=1}^{L_{sd}}\alpha_{sd,l}\cdot e_{sd,k-\tau_{sd,l}}+n_{sd,k}$$

where $L_{sr(sd)}$ is the number of channel paths. $\alpha_{sr(sd),l}$ is the *lth* channel path loss coefficient. $e_{sr,k-\tau_{sr,l}}$ and $e_{sd,k-\tau_{sd,l}}$ are the transmitted signals $e_{sr(sd)}$ after passing through the multipath Rayleigh fading channels. $n_{sr(sd),k}$ is the AWGN with zero mean and variance $N_{0,sr(sd)}/2$ for the S→R(D) link. $\tau_{sr(sd),l}$ represents the latency time of the transmitted signal $e_{sr(sd)}$ after passing through the multipath Rayleigh fading channels. *k* represents the *kth* value of the transmission period.

The decision variable of the S→R link can be denoted as

$$Z_{sr}=\sum_{j=1}^{J}\sum_{k=1}^{U}y_{sr,k}\cdot y_{sr,k+nU}$$

$$=\sum_{j=1}^{J}\sum_{k=1}^{U}\left(\sum_{l=1}^{L_{sr}}\alpha_{sr,l}\cdot e_{sr,k-\tau_{sr,l}}+n_{sr,k}\right)\cdot \left(\sum_{l=1}^{L_{sr}}\alpha_{sr,l}\cdot e_{sr,k+nU-\tau_{sr,l}}+n_{sr,k+nU}\right)$$

(4) $$=\sum_{j=1}^{J}\sum_{k=1}^{U}\left(b_{n}\sum_{l=1}^{L_{sr}}\alpha_{sr,l}^{2}\cdot x_{k-\tau_{sr,l}}^{2}+\sum_{l=1}^{L_{sr}}\alpha_{sr,l}\cdot x_{k-\tau_{sr,l}}\cdot n_{sr,k+nU}+b_{n}\sum_{l=1}^{L_{sr}}\alpha_{sr,l}\cdot x_{k-\tau_{sr,l}}\cdot n_{sr,k}\;+n_{sr,k}\cdot n_{sr,k+nU}\right)\;\;\;\;\;\;\;\;\;\;\;\;\;\;$$

Therefore, if $Z_{sr}>0$, the polarity-modulated symbol $b_{n}$ is estimated as $b_{n}=+1$; otherwise, $b_{n}$ is estimated as $b_{n}=-1$.

The decision variable of the S→D link can be denoted as

$$Z_{sd}=\sum_{j=1}^{J}\sum_{k=1}^{U}y_{sd,k}\cdot y_{sd,k+nU}\;$$

$$=\sum_{J=1}^{J}\sum_{k=1}^{U}\left(\sum_{l=1}^{L_{sd}}\alpha_{sd,l}\cdot e_{sd,k-\tau_{sd,l}}+n_{sd,k}\right)\cdot \left(\sum_{l=1}^{L_{sd}}\alpha_{sd,l}\cdot e_{sd,k+nU-\tau_{sd,l}}+n_{sd,k+nU}\right)$$

(5) $$\;\;\;\;\;\;\;\;\;=\sum_{j=1}^{J}\sum_{k=1}^{U}\left(b_{n}\sum_{l=1}^{L_{sd}}\alpha_{sd,l}^{2}\cdot x_{k-\tau_{sd,l}}^{2}+\sum_{l=1}^{L_{sd}}\alpha_{sd,l}\cdot x_{k-\tau_{sd,l}}\cdot n_{sd,k+nU}\;+b_{n}\sum_{l=1}^{L_{sd}}\alpha_{sd,l}\cdot x_{k-\tau_{sd,l}}\cdot n_{sd,k}+n_{sd,k}\cdot n_{sd,k+nU}\right)\;\;\;\;\;$$

### 2.3. R-D Link

**Figure 4:**
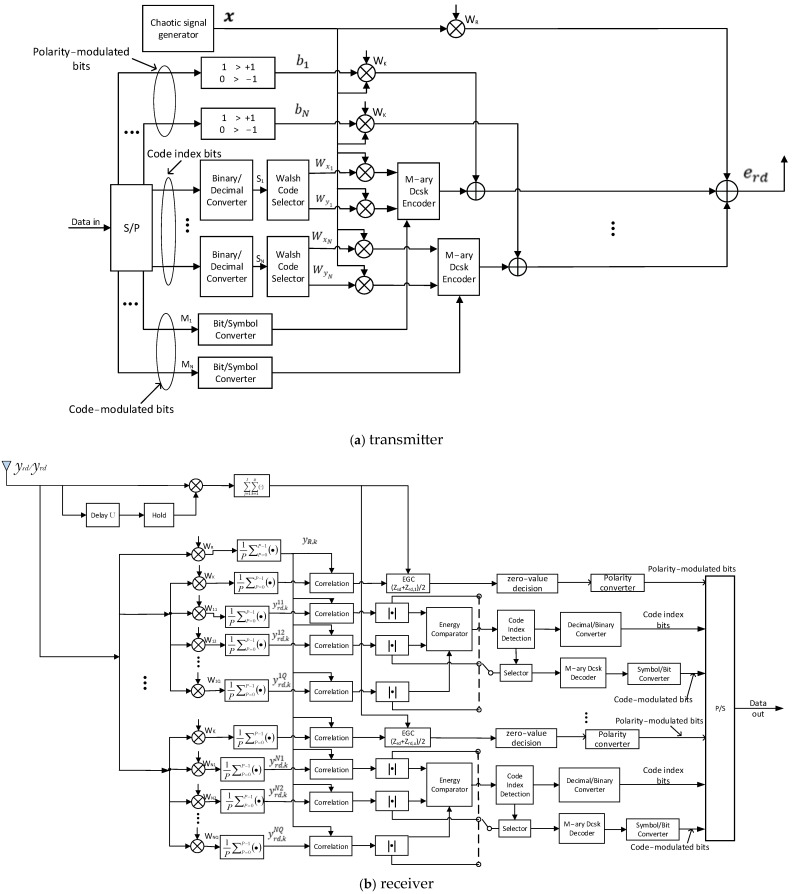
The block diagram of the MCIM-MDCSK modulation scheme.

The MCIM-MDCSK modulation scheme is adopted at R. The block diagram of the MCIM-MDCSK modulation scheme is shown in Figure 4. The transmitted signal at R can be expressed as

(6) $$e_{rd}=W_{R}⨂x+c_{n}\cdot W_{x_{n}}⨂x+d_{n}\cdot W_{y_{n}}⨂x+b_{n}\cdot W_{k}⨂x$$

In Equation (6), $W_{R}$ is the reference Walsh code. $W_{k}$ is utilized for the polarity-modulated bits. $W_{x_{n}}$ and $W_{y_{n}}$ are the outputs of the Walsh code selector, where $x_{n}=2S_{n}+1$ and $y_{n}=2S_{n}+2$. Here, $S_{n}$ represents the code index symbols. The length of Walsh codes is P. $c_{n}$ and $d_{n}$ are the in-phase and quadrature components of the $n^{th}$ M-ary constellation symbol $M_{n}=c_{n}+jd_{n}$.

The reference signal received at D can be expressed as

(7) $$y_{R,k}=\frac{1}{P}\sum_{p=0}^{P-1}W_{R,p+1}\left[\sum_{l=1}^{L_{rd}}\alpha_{rd,l}{\cdot W}_{R,p+1}\cdot x_{k-\tau_{rd,l}}+n_{rd,k}\right]=\sum_{l=1}^{L_{rd}}\alpha_{rd,l}\cdot x_{k-\tau_{rd,l}}+n_{R,k}$$

where

(8) $$n_{R,k}=\frac{1}{P}\sum_{p=0}^{P-1}W_{R,p+1}{\cdot n}_{rd,k}$$

The *nth* information signal received at D can be expressed as

$$y_{rd,k}^{nq}=\frac{1}{P}\sum_{p=0}^{P-1}W_{nq,p+1}\left[\sum_{l=1}^{L_{rd}}\alpha_{rd,l}{\cdot (c_{n}W}_{x_{n},p+1}\cdot x_{k-\tau_{rd,l}}+{d_{n}W}_{y_{n},p+1}\cdot x_{k-\tau_{rd,l}}+{b_{n}W}_{k,p+1}\;\cdot x_{k-\tau_{rd,l}})+n_{rd,k}\right]$$

(9) $$=\left\{\begin{array}{c} \sum_{l=1}^{L_{rd}}\alpha_{rd,l}\cdot c_{n}\cdot x_{k-\tau_{rd,l}}+n_{x,k},\;\;\;\;\;q=x_{n}\\ \sum_{l=1}^{L_{rd}}\alpha_{rd,l}\cdot d_{n}\cdot x_{k-\tau_{rd,l}}+n_{y,k},\;\;\;\;\;q=y_{n}\\ n_{q,k},\;\;\;\;\;\;\;\;\;\;\;\;\;\;\;\;\;\;\;\;\;\;\;\;\;\;\;\;\;\;\;\;\;\;\;\;\;\;\;\;\;\;\;\;\;\;\;\;\;\;\;\;q\neq x_{n}\;and\;\;q\neq y_{n}\\ \sum_{l=1}^{L_{rd}}\alpha_{rd,l}\cdot b_{n}\cdot x_{k-\tau_{rd,l}}+n_{W_{k}},\;\;\;\;\;W_{nq}=W_{k} \end{array}\right.$$

where

(10) $$n_{x,k}=\frac{1}{P}\sum_{p=0}^{P-1}W_{nq,p+1}\cdot n_{rd,k},\;\;\;\;\;q=x_{n}$$

(11) $$n_{y,k}=\frac{1}{P}\sum_{p=0}^{P-1}W_{nq,p+1}\cdot n_{rd,k},\;\;\;\;\;q=y_{n}$$

(12) $$n_{q,k}=\frac{1}{P}\sum_{p=0}^{P-1}W_{nq,p+1}\cdot n_{rd,k},\;\;q\neq x_{n}\;and\;q\neq y_{n}$$

(13) $$n_{W_{k}}=\frac{1}{P}\sum_{p=0}^{P-1}W_{k,p+1}\cdot n_{rd,k},\;\;\;\;\;W_{nq}=W_{k}$$

The decision variable of the nth signal in the R→D link can be denoted as

$$Z_{nq}=\sum_{k=1}^{U}y_{R,k}\cdot y_{rd,k}$$

(14) $$=\left\{\begin{array}{c} \sum_{k=1}^{U}\left(\sum_{l=1}^{L_{rd}}\alpha_{rd,l}\cdot x_{k-\tau_{rd,l}}+n_{R,k}\right)\cdot \left(\sum_{l=1}^{L_{rd}}\alpha_{rd,l}\cdot {c_{n}\cdot x}_{k-\tau_{rd,l}}+n_{x,k}\right),\;\;\;\;\;\;\;\;\;\;\;\;\;\;\;q=x_{n}\\ \sum_{k=1}^{U}\left(\sum_{l=1}^{L_{rd}}\alpha_{rd,l}\cdot x_{k-\tau_{rd,l}}+n_{R,k}\right)\cdot \left(\sum_{l=1}^{L_{rd}}\alpha_{rd,l}\cdot {d_{n}\cdot x}_{k-\tau_{rd,l}}+n_{y,k}\right),\;\;\;\;\;\;\;\;\;\;\;\;\;\;\;\;\;\;q=y_{n}\\ \sum_{k=1}^{U}\left(\sum_{l=1}^{L_{rd}}\alpha_{rd,l}\cdot x_{k-\tau_{rd,l}}+n_{R,k}\right)\cdot n_{q,k},\;\;\;\;\;\;\;\;\;\;\;\;\;\;\;\;\;\;\;\;\;\;\;\;\;\;\;\;\;\;\;\;\;\;\;\;\;\;\;\;\;\;\;\;\;q\neq x_{n}\;and\;\;q\neq y_{n}\\ \sum_{k=1}^{U}\left(\sum_{l=1}^{L_{rd}}\alpha_{rd,l}\cdot x_{k-\tau_{rd,l}}+n_{R,k}\right)\cdot \left(\sum_{l=1}^{L_{rd}}\alpha_{rd,l}\cdot b_{n}\cdot x_{k-\tau_{rd,l}}+n_{W_{k}}\right)=Z_{W_{k}},\;W_{nq}=W_{k} \end{array}\right.$$

Finally, by utilizing an equal-gain combiner (EGC) to process the decision metrics of the S→D and R→D links, the polarity-modulated symbol can be estimated as

(15) $$\hat{b_{n}}=\left\{\begin{array}{c} +1,\;\;\;\;\;\;\;\;{(Z_{sd}+Z}_{W_{k}})/2>0\\ -1,\;\;\;\;\;\;\;\;\;\;\;\;\;\;\;\;\;\;\;\;\;\;\;\;\;\;\;\;\;\;\;\;others \end{array}\right.$$

The code index bits can be estimated as

(16) $$\hat{s_{n}}=\arg\underset{q=1,\ldots Q}{\max}\left(\left|Z_{nq}\right|\right)$$

By using the Euclidean distance decision method [22], the M-ary constellation symbols corresponding to the maximum energy in each group can be estimated. Then the code-modulated bits can be recovered by the symbol-to-bit converters.

## 3. Performance Analysis

### 3.1. BER

In the MCIM-SR-DCSK-CC system, the overall BER is concluded by the BER of the polarity-modulated bits, the BER of the code index bits, and the BER of the code-modulated bits. Therefore, the BER expression of the proposed system can be obtained as

(17) $$P_{sys}=\frac{1}{m_{ci}+m_{cm}+1}P_{pm}+\frac{m_{ci}}{m_{ci}+m_{cm}+1}P_{ci}+\frac{m_{cm}}{m_{ci}+m_{cm}+1}P_{cm}\;\;$$

where $P_{pm}$, $P_{ci}$, and $P_{cm}$ are defined as the BERs of the polarity-modulated bits, the code index bits, and the code-modulated bits, respectively. $m_{ci}$ is the number of the code index bits. $m_{cm}$ is the number of the code-modulated bits.

#### 3.1.1. Derivation of $P_{ci}$ and $P_{cm}$

$P_{ci}$ and $P_{cm}$ are related to the error probability of the Walsh code detection $P_{ed}$. The probability of detecting any of the Walsh-encoded $2^{m_{ci}}-1$ error row vectors is the same, and it equals $\frac{1}{2^{m_{ci}}-1}$. The expectation of the number of wrong code index bits is [16,34]

(18) $$D=\sum_{i=1}^{m_{ci}}i\cdot \frac{\left(\begin{array}{c} m_{ci}\\ i \end{array}\right)}{2^{m_{ci}}-1}\;$$

where $\left(\begin{array}{c} m_{ci}\\ i \end{array}\right)=\frac{m_{ci}!}{i!\left(m_{ci}-i\right)!}$. Thus, the BER of the code index bits can be calculated as [16,33]

(19) $$P_{ci}=\frac{D}{m_{ci}}P_{ed}$$

For the BER of the code-modulated bits, the error could be caused by two separate mechanisms. On the one hand, the code index detection is correct, but the code-modulated bits are recovered incorrectly. On the other hand, there is an error in the code index detection. Therefore, the BER of the code-modulated bits can be given as [16,17,18,34]

(20) $$P_{cm}=P_{e}\left(1-P_{ed}\right)+\frac{1}{2}P_{ed}$$

where $P_{e}$ is the BER of the MDCSK modulation.

To calculate $P_{ed}$, the mean of $Z_{nq}$ is obtained as

(21) $${E[Z}_{nq}]=\left\{\begin{array}{c} c_{n}\sum_{l=1}^{L_{rd}}\alpha_{rd,l}^{2}\frac{E_{s}}{(N+1)U},\;\;\;\;\;\;\;q=x_{n}\\ d_{n}\sum_{l=1}^{L_{rd}}\alpha_{rd,l}^{2}\frac{E_{s}}{(N+1)U},\;\;\;\;\;\;\;q=y_{n}\\ \;0,\;\;\;\;\;\;\;\;\;\;\;\;\;\;\;\;\;\;\;\;\;q\neq x_{n}\;and\;\;q\neq y_{n}\\ b_{n}\sum_{l=1}^{L_{rd}}\alpha_{rd,l}^{2}\frac{E_{s}}{(N+1)U},\;\;\;\;W_{nq}=W_{k} \end{array}\;\right.$$

$c_{n}$ and $d_{n}$ are the in-phase and quadrature components of the $n^{th}$ M-ary constellation symbol $M_{n}=c_{n}+jd_{n}$. $c_{n}$ and $d_{n}$ satisfy $c_{n}^{2}+d_{n}^{2}=1$. There are M different combinations of values for $c_{n}$ and $d_{n}$. The system performance indicators obtained in the case of different combinations of values are the same. To simplify the analysis, we choose one of them to analyze the system performance. Therefore, we assume $c_{n}=d_{n}=\frac{\sqrt{2}}{2}$. $b_{n}$ is the polarity-modulated symbol. There are two values for $b_{n}$: +1 and −1. We assume $b_{n}=+1$ for the convenience of the theoretical derivation. Thus, ${E[Z}_{nq}]$ can be simplified as

(22) $${E[Z}_{nq}]=\left\{\begin{array}{c} \frac{\sqrt{2}}{2}\sum_{l=1}^{L_{rd}}\alpha_{rd,l}^{2}\frac{E_{s}}{\left(N+1\right)U}=\mu_{1},\;\;\;\;\;q=x_{n}\;or\;\;q=y_{n}\\ \;0,\;\;\;\;\;\;\;\;\;\;\;\;\;\;\;\;\;\;\;\;\;\;\;\;\;\;\;\;\;\;\;\;\;\;\;\;\;\;\;\;\;\;\;\;\;\;\;\;\;\;q\neq x_{n}\;and\;\;q\neq y_{n}\\ \sum_{l=1}^{L_{rd}}\alpha_{rd,l}^{2}\frac{E_{s}}{\left(N+1\right)U},\;\;\;\;\;\;\;\;\;\;\;\;\;\;\;\;\;\;\;\;\;\;\;\;\;\;\;\;\;\;\;\;\;\;\;\;\;\;\;W_{nq}=W_{k} \end{array}\right.\;$$

Then, the variance of $Z_{nq}$ is obtained as

(23) $${Var[Z}_{nq}]=\left\{\begin{array}{c} \frac{3}{4}\sum_{l=1}^{L_{rd}}\alpha_{rd,l}^{2}\frac{E_{s}N_{0,rd}}{\left(N+1\right)U}+\frac{N_{0,rd}^{2}}{4}U=\sigma_{1}^{2},\;\;\;\;q=x_{n}\;or\;q=y_{n}\\ \frac{1}{2}\sum_{l=1}^{L_{rd}}\alpha_{rd,l}^{2}\frac{E_{s}N_{0,rd}}{\left(N+1\right)U}+\frac{N_{0,rd}^{2}}{4}U=\sigma_{2}^{2},\;\;\;q\neq x_{n}\;and\;q\neq y_{n}\\ \sum_{l=1}^{L_{rd}}\alpha_{rd,l}^{2}\frac{E_{s}N_{0,rd}}{\left(N+1\right)U}+\frac{N_{0,rd}^{2}}{4}U=\sigma_{3}^{2},\;\;\;\;\;W_{nq}=W_{k} \end{array}\right.$$

where $E_{s}=\left(1+N\right)UE[x_{k}^{2}]$ is defined as the average symbol energy of the proposed system. We set $\gamma_{rd}=\sum_{l=1}^{L_{rd}}\alpha_{rd,l}^{2}\frac{E_{s}}{N_{0,rd}}$, ${Var[Z}_{nq}]$ can be simplified as

(24) $${Var[Z}_{nq}]=\left\{\begin{array}{c} \sum_{l=1}^{L_{rd}}\alpha_{rd,l}^{2}E_{s}N_{0,rd}(\frac{3}{4(N+1)U}+\frac{U}{4\gamma_{rd}})=\sigma_{1}^{2},\;\;\;\;\;\;\;q=x_{n}\;\;or\;\;q=y_{n}\\ \sum_{l=1}^{L_{rd}}\alpha_{rd,l}^{2}E_{s}N_{0,rd}(\frac{1}{2(N+1)U}+\frac{U}{4\gamma_{rd}})=\sigma_{2}^{2},\;\;\;\;\;\;\;q\neq x_{n}\;and\;\;q\neq y_{n}\\ \sum_{l=1}^{L_{rd}}\alpha_{rd,l}^{2}E_{s}N_{0,rd}(\frac{1}{\left(N+1\right)U}+\frac{U}{4\gamma_{rd}})=\sigma_{3}^{2},\;\;\;\;\;\;\;\;\;\;\;\;\;\;\;\;\;\;\;\;\;\;\;\;\;W_{nq}=W_{k} \end{array}\right.$$

where $\gamma_{rd}$ is the instantaneous signal-to-noise ratio (SNR) of the R→D link.

We assume that the recovered code index symbol of the $n^{th}$ path is $\hat{s_{n}}=\hat{q},q=x_{n}\;or\;q=y_{n}$. The random variables $\left|Z_{n\hat{q}}\right|$ and $\left|Z_{nq}\right|$ follow identical folded normal distribution [21,32]. Thus, the probability density function (PDF) of $\left|Z_{n\hat{q}}\right|$ and the cumulative distribution function of $\left|Z_{nq}\right|$ are formulated as

(25) $$f_{\left|Z_{n\hat{q}}\right|}\left(r\right)=\frac{1}{\sqrt{2\pi \sigma_{1}^{2}}}[\exp\left(-\frac{{\left(r-\mu_{1}\right)}^{2}}{2\sigma_{1}^{2}}\right)+\exp\left(-\frac{{\left(r+\mu_{1}\right)}^{2}}{2\sigma_{1}^{2}}\right)]\;\;$$

(26) $$F_{\left|Z_{nq}\right|}\left(r\right)=\mathrm{erf}\left(\frac{r}{\sqrt{2\sigma_{2}^{2}}}\right)$$

Then, the mean and the variance of $\left|Z_{n\hat{q}}\right|$ can be given as

(27) $$\mu_{\left|Z_{n\hat{q}}\right|}=\sqrt{\frac{2}{\pi }}\sigma_{1}\exp\left(-\frac{{\mu_{1}}^{2}}{2\sigma_{1}^{2}}\right)-\mu_{1}\mathrm{erf}\left(-\sqrt{\frac{{\mu_{1}}^{2}}{2\sigma_{1}^{2}}}\right)$$

(28) $$\sigma_{\left|Z_{n\hat{q}}\right|}^{2}={\mu_{1}}^{2}+\sigma_{1}^{2}-\mu_{\left|Z_{n\hat{q}}\right|}^{2}$$

It is assumed that $Y_{1}=max\{\left|Z_{nq}\right|\}$ and q=1,2,…,Q−1. The conditional error probability of the Walsh code detection is calculated by

(29) $$P_{ed}\left(e\left|\gamma_{rd}\right.\right)=\int_{0}^{\infty }\left[1-\mathrm{Pr}\left\{Y_{1}\leq x\right\}\right]f_{\left|Z_{n\hat{q}}\right|}\left(x\right)dx=\int_{0}^{\infty }\left[1-\prod_{q=1}^{Q-1}Pr\{Z_{nq}\leq x\}\right]f_{\left|Z_{n\hat{q}}\right|}\left(x\right)\;=\frac{1}{\sqrt{2\pi \sigma_{\left|Z_{n\hat{q}}\right|}^{2}}}\int_{0}^{\infty }\left\{{1-\left[\mathrm{erf}\left(\frac{x}{\sqrt{2\sigma_{2}^{2}}}\right)\right]}^{Q-1}\right\}\cdot \{\exp\left[-\frac{{\left(x+\mu_{\left|Z_{n\hat{q}}\right|}\right)}^{2}}{2\sigma_{\left|Z_{n\hat{q}}\right|}^{2}}\right]\;\;\;\;\;\;\;+exp[-\frac{{\left(x-\mu_{\left|Z_{n\hat{q}}\right|}\right)}^{2}}{2\sigma_{\left|Z_{n\hat{q}}\right|}^{2}}]\}dx\;\;\;\;\;\;\;\;\;\;\;\;\;\;\;\;\;\;\;\;$$

It is assumed that $L_{v},v\in \{sr,sd,rd\}$ channel coefficients are independent and identically distributed (i.i.d) Rayleigh-fading channels. Simultaneously, it is assumed that the average power gains of the $L_{v}$ are equal. The PDF of $\gamma_{v},v\in \{sr,sd,rd\}$ can be expressed as [21,32]

(30) $$f\left(\gamma_{v}\right)=\frac{\gamma_{v}^{L_{v}-1}}{\left(L_{v}-1\right)!{\bar{\gamma }}^{L_{v}}}\exp\left(-\frac{\gamma_{v}}{\bar{\gamma }}\right)$$

where the average SNR is $\bar{\gamma }=\frac{E_{s}}{N_{0,v}}E\left[\alpha_{v,l}^{2}\right],l\in \{1,2,\ldots ,L_{v}\}$ with $\sum_{l=1}^{L_{v}}{E[\alpha }_{v,l}^{2}]=1$.

Therefore, the average error probability of the Walsh code detection can be given as

(31) $$P_{ed}=\int_{0}^{\infty }P_{ed}\left(e\left|\gamma_{rd}\right.\right)f\left(\gamma_{rd}\right)d\gamma_{rd}$$

#### 3.1.2. Derivation of $P_{pm}$

The modulated bit is transmitted from S, forwarded by R, and then recovered at D. The first step is to analyze the error probability of the DF protocol, which is determined by the erroneous detection of SR-DCSK at R. To obtain the decision metric (i.e., $Z_{sr}$) for SR-DCSK detection, the mean and the variance of $Z_{sr}$ can be expressed as

(32) $${E[Z}_{sr}]=b_{n}\sum_{l=1}^{L_{sr}}\alpha_{sr,l}^{2}\frac{NE_{s}}{(N+1)U}\;\;\;$$

(33) $${V_{ar}[Z}_{sr}]=\sum_{l=1}^{L_{sr}}\alpha_{sr,l}^{2}\frac{NE_{s}N_{0,sr}}{(N+1)U}+\frac{N_{0,sr}^{2}}{4}NU$$

Based on the central limit theorem, the decision metric $Z_{sr}$ follows a normal distribution [32], and the conditional error probability of the DF protocol can be calculated as

(34) $$P_{df}\left(e\left|\gamma_{sr}\right.\right)=\frac{1}{2}erfc\left\{{\left[\frac{2{V_{ar}[Z}_{sr}]}{{{\{E[Z}_{sr}]\}}^{2}}\right]}^{-\frac{1}{2}}\right\}=\frac{1}{2}erfc\left\{{\left[\frac{2(N+1)U}{N\gamma_{sr}}+\frac{{\left(N+1\right)}^{2}U}{2N\gamma_{sr}^{2}}\right]}^{-\frac{1}{2}}\right\}$$

where $\gamma_{sr}=\sum_{l=1}^{L_{sr}}\alpha_{sr,l}^{2}\frac{E_{s}}{N_{0,sr}}$ is the instantaneous SNR of the S→R link.

Thus, the average error probability of the DF protocol can be obtained as

(35) $$P_{df}=\int_{0}^{\infty }P_{df}\left(e\left|\gamma_{sr}\right.\right)f\left(\gamma_{sr}\right)d\gamma_{sr}\;$$

At D, EGC is utilized to obtain the decision metric $Z_{egc}$ of the polarity-modulated bit. Indeed, the decision metric can be computed by those of the S→D and R→D links, i.e., $Z_{egc}=(Z_{sd}+Z_{rd})/2$. When the Walsh code detection is accurate, the decision metric of the R→D link can be denoted as $Z_{rd}=Z_{n\hat{q}}$, and the resulting decision metric can be expressed as $Z_{egc}=(Z_{sd}+Z_{n\hat{q}})/2$. In other cases, $Z_{rd}=Z_{nq}(\left\{q\in 1,2,\ldots ,Q\right\}andq\neq \hat{q}$), leading to the decision metric of $Z_{egc}=(Z_{sd}+Z_{nq})/2$.

When calculating the BER of the polarity-modulated bit, there are four possible situations that can arise in the MCIM-SR-DCSK-CC system. Specifically, these four situations are defined as follows:

Case 1: Correct Detection at Relay and Correct Detection at Destination

In this case, both the relay and the destination correctly detect the polarity-modulated bit. This is the ideal scenario where no errors occur in the transmission.

(36) $$P_{r}\left(\Theta =1\right)=\left(1-P_{df}\right)\left(1-P_{ed}\right)$$

Case 2: Correct Detection at Relay and Incorrect Detection at Destination

Here, the relay correctly detects the polarity-modulated bit, but the destination makes an error in detection.

(37) $$P_{r}\left(\Theta =2\right)=P_{df}\left(1-P_{ed}\right)$$

Case 3: Incorrect Detection at Relay and Correct Detection at Destination

In this scenario, the relay makes an error in detecting the polarity-modulated bit, but the destination correctly detects it. This could happen if the S→R link is impaired but the R→D link is clear.

(38) $$P_{r}\left(\Theta =3\right)=P_{ed}\left(1-P_{df}\right)$$

Case 4: Incorrect Detection at Relay and Incorrect Detection at Destination

This is the worst-case scenario where both the relay and the destination incorrectly detect the polarity-modulated bit.

(39) $$P_{r}\left(\Theta =4\right)=P_{ed}P_{df}$$

Therefore, the BER of the polarity-modulated bit can be obtained as

(40) $$P_{pm}=\sum_{\Omega =1}^{4}P_{r}\left(\Theta =\Omega \right)P\left(\left.e\right|\Theta =\Omega \right)$$

Additionally, the conditional BERs of the four cases are obtained in Equations (41)–(43), where $\gamma_{sd}=\sum_{l=1}^{L_{sd}}\alpha_{sd,l}^{2}\frac{E_{s}}{N_{0,sd}}$ corresponds to the instantaneous SNR of the S→D link.

(41) $$P\left(\left.e\right|\Theta =1\right)=\frac{1}{2}\int_{0}^{\infty }\int_{0}^{\infty }erfc\left({\left[\frac{\left(1+N\right)}{\sqrt{N}}\cdot \frac{\sqrt{\gamma_{sd}+\gamma_{rd}+U}}{\left(\gamma_{sd}+\gamma_{rd}\right)}\right]}^{-1}\right)f\left(\gamma_{sd}\right)f\left(\gamma_{rd}\right)d\gamma_{sd}d\gamma_{rd}\;$$

(42) $$P\left(\left.e\right|\Theta =2\right)=\frac{1}{2}\int_{0}^{\infty }\int_{0}^{\infty }erfc\left({\left[\frac{\left(1+N\right)}{\sqrt{N}}\cdot \frac{\sqrt{\gamma_{sd}+\gamma_{rd}+U}}{\left(\gamma_{sd}-\gamma_{rd}\right)}\right]}^{-1}\right)f\left(\gamma_{sd}\right)f\left(\gamma_{rd}\right)d\gamma_{sd}d\gamma_{rd}\;$$

(43) $$P\left(\left.e\right|\Theta =3\right)=P\left(\left.e\right|\Theta =4\right)\;\;\;\;\;\;\;\;\;\;=\frac{1}{2}\int_{0}^{\infty }\int_{0}^{\infty }erfc([\frac{\left(1+N\right)}{\sqrt{N}}\;\;\;\;\;\;\;\;\;\;\cdot \frac{\sqrt{\gamma_{sd}+\frac{\gamma_{rd}}{N+1}+U}}{\gamma_{sd}}{]}^{-1})f\left(\gamma_{sd}\right)f\left(\gamma_{rd}\right)d\gamma_{sd}d\gamma_{rd}\;\;\;\;\;\;\;\;\;\;\;\;$$

Therefore, by substituting Equations (36)–(39) and Equations (41)–(43) into Equation (40), the BER of the polarity-modulated bit can be derived.

### 3.2. Transmission Throughput

The normalized throughput is defined as the ratio of the successfully received bits/second of a given DCSK system to the transmitted bits/second of the proposed MCIM-SR-DCSK-CC system [1,32]. The proposed MCIM-SR-DCSK-CC system can send N information bit streams per transmission period, i.e., $\bar{T}=\left(U+RU\right){(1+m_{ci}+m_{cm})T}_{c}\times 2$. $N_{p}=1+m_{ci}+m_{cm}$ represents the number of bits transmitted per group.

Thus, the normalized throughput can be calculated as [32]

(44) $$R_{t}=\frac{\left[{\left(1-P_{sys}^{t}\right)}^{N_{p}}N_{p}\right]/\bar{T_{t}}}{N_{p}/\bar{T}}=\frac{{\left(1-P_{sys}^{t}\right)}^{N}\bar{T}}{\bar{T_{t}}}$$

where $P_{sys}^{t}$ and $\bar{T_{t}}$ represent the average BER and the time required to transmit N information bit streams for a given system t∈{CIM−SR−DCSK−CC,MCIM−SR−DCSK−CC}, respectively. The time durations required for transmitting N information bit streams for the CIM−SR−DCSK−CC system is $\left(U+RU\right)\frac{1+m_{ci}}{1+m_{ci}+m_{cm}}N_{p}T_{c}\times 2$.

### 3.3. Efficiency Discussions

**Figure 5:**
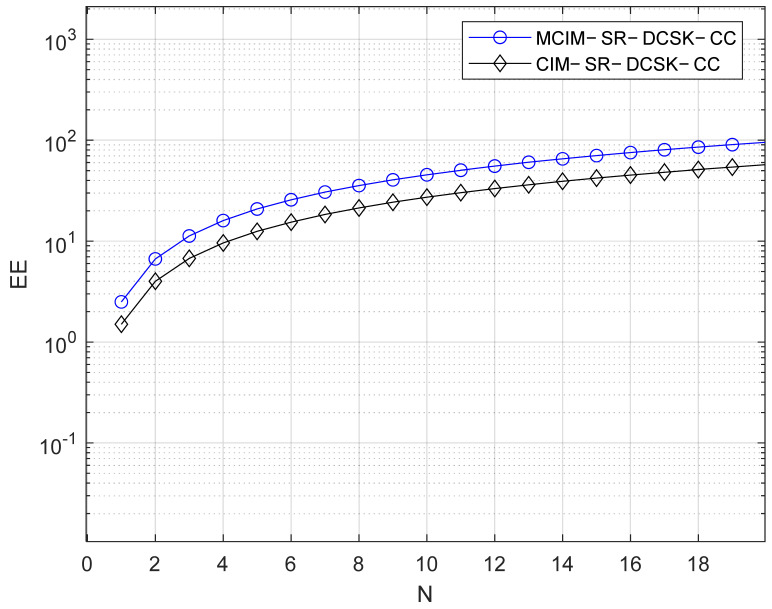
EE comparisons with the systems.

**Figure 6:**
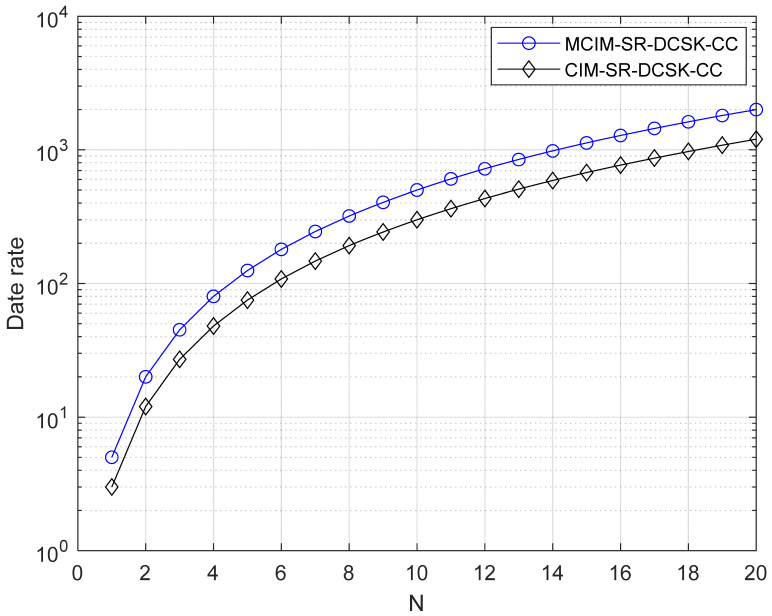
Data rate comparisons with the systems.

Table 1 displays the comparisons of EE and the data rate, which are discussed in this section. For visual representation, the comparisons of EE and the data rate are also given in Figure 5 and Figure 6, respectively. N is the number of parallel groups. Here, we set M = 4, i.e., $m_{ci}=m_{cm}=2$. The better performance in EE and the data rate are demonstrated by the proposed system.

To research the EE, we calculate the transmitted data-energy-to-bit-energy ratio (DBR) [10]:

(45) $$DBR=\frac{E_{data}}{E_{b}}$$

where $E_{data}$ is the energy to transmit the data, and $E_{b}$ is the transmitted bit energy.

### 3.4. Computational Complexity

The assessment of the computational complexity is dependent on the number of multiplication operations for spreading/de-spreading during one symbol duration [35,36,37,38]. In the source node S of the proposed system, NJ×U multiplication operations are required. In the relay node R of the proposed system, NJ×U+N(N+3)×U multiplication operations are necessary. In the destination node D of the proposed system, NJ×U+2NQ×U multiplication operations are needed. Therefore, the computational complexity of the proposed system is $3NJ\times U+N\left(N+3\right)\times U+2NQ\times U$. Likewise, the computational complexity of CIM-SR-DCSK-CC is 3J×U+2×U+Q×U. Obviously, the proposed system shows higher computational complexity than the CIM-SR-DCSK-CC system.

### 3.5. Memory Requirement

In our system, the memory requirements mainly come from the following aspects:

① Data caching:

In relay nodes and terminal devices, some of the transmitted data need to be cached to support multi-hop transmission and related processing operations. Specifically, each relay node needs to store the data from the previous hop until they are forwarded to the next hop. Assuming that the amount of data processed by each relay node is D bytes and there is one relay node in the system, the total memory requirement of the relay nodes is D bytes.

② Coding information storage:

In order to realize additional coded modulation functions, coding parameters and related code word information need to be stored. For orthogonal codes such as Walsh codes, the memory requirement is related to the code length and the number of code words. For example, for a Walsh code of length P, it is necessary to store $P\times \log_{2}P$ bits of code word information.

③ Correlation computation storage:

When multiple correlation computations are performed, the intermediate results need to be stored temporarily. Assuming that each correlation calculation involves $U^{2}$ data points, each calculation unit needs to store $U^{2}$ bytes of intermediate results.

We quantitatively analyzed memory requirements to assess their impact on system resources. The details are as follows:

① Relay node memory requirement: assuming that the amount of data handled by each relay node is 1024 bytes (1 KB) and there is one relay node in the system, the total memory requirement of the relay nodes is 1 KB.

② Coding information storage: for a Walsh code with length 64, it needs to store $64*\log_{2}64=384bits,$ i.e., 48 bytes.

③ Correlation calculation storage: assuming that each correlation calculation involves 200 data points and each data point is 1 byte, then each calculation unit needs to store an additional 200 bytes of intermediate results.

The above memory requirements are acceptable in modern communication systems. For example, modern relay nodes are typically equipped with large memory capacities (e.g., 128 MB or more), so the 1 KB storage requirement represents a very small fraction (about 0.0007%) of their total storage capacity.

## 4. Simulation Results and Discussions

In this part, the performance analysis of the proposed MCIM-SR-DCSK-CC system over the AWGN channel and the multipath Rayleigh fading channel is shown. We adopt the three-path Rayleigh fading channel, which is usually utilized in many chaos-based communication systems. The delays of the propagation paths in this three-path Rayleigh fading channel are τ_1_ = 0, τ_2_ = 2, and τ_3_ = 3. And the power gains are equal with $E\left[\alpha_{1}^{2}\right]=E\left[\alpha_{2}^{2}\right]=E\left[\alpha_{3}^{2}\right]=\frac{1}{3}$. Additionally, the channel models used in the S→R link and the S→D and R→D links are the same.

When the length of the Walsh codes increases, the noise variance will decrease. Then, the BER performance improves. Therefore, the BER performance improves as the length of the Walsh codes increases. However, the system complexity increases when the length of the Walsh codes increases. But the BER performance and the bit rate improve. Therefore, the trade-off between performance and system complexity can be selected according to actual needs. In this paper, we consider both not too much complexity and better system performance, so we set the length of the Walsh codes to 16 or 64.

**Figure 7:**
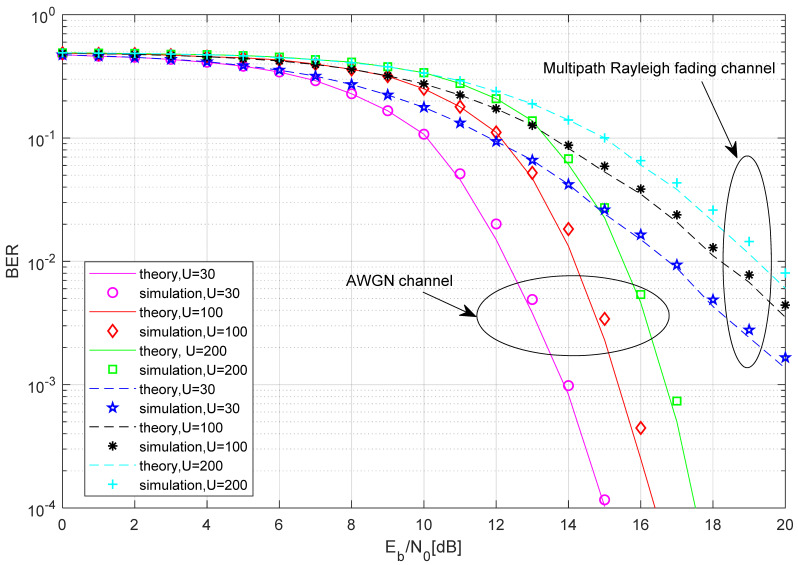
The BER performance comparison between the simulated and the theoretical results of the proposed MCIM-SR-DCSK-CC system.

In Figure 7, the BER performance comparison between the simulated and the theoretical results of the proposed MCIM-SR-DCSK-CC system is given with M=4,N=10,J=4,P=16, and different U. Intuitively, the consistency between the simulation results and the theoretical results serves as additional evidence for the correctness and effectiveness of the theoretical analysis. As the length of the chaotic signal shortens, the BER performance of the proposed system gets better. This is because a longer chaotic signal would lead to a more pronounced noise component in the noise–noise correlation term, thereby degrading the BER performance.

**Figure 8:**
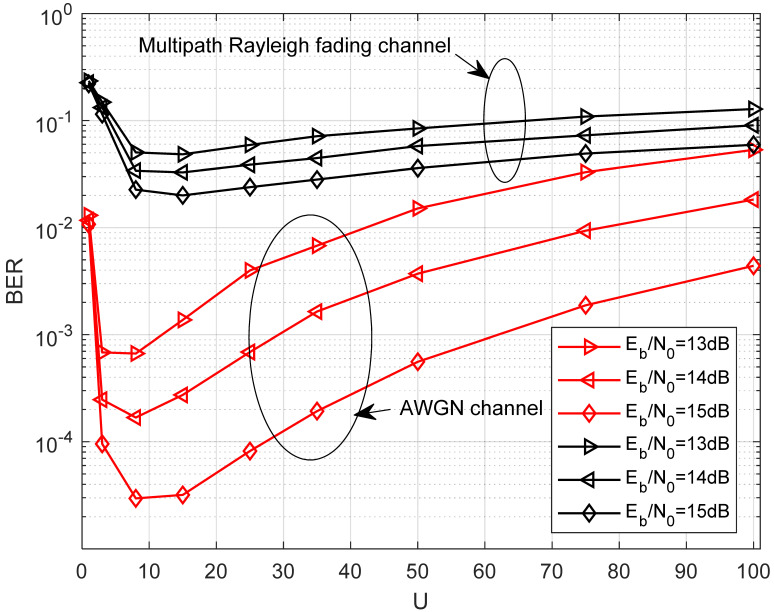
BER performance of the proposed system versus the length of the chaotic signal over the AWGN channel and multipath Rayleigh fading channel with P=16,M=4,J=4,N=10.

In Figure 8, the effect of different lengths of the chaotic signal on the BER of the proposed system over the AWGN channel and multipath Rayleigh fading channel is demonstrated when $\frac{E_{b}}{N_{0}}$ is fixed. It is evident that the BER initially decreases with U, hits an optimal point, and subsequently begins to increase. It indicates that an optimal choice of U exists in the proposed system. When the length of the chaotic signal increases, the noise component involved in the noise–noise correlation term becomes more significant, resulting in a poor BER performance. However, the computational complexity of the system will improve when the length of the chaotic signal increases. Furthermore, it can be seen from Figure 8 that the optimal selection of U is approximately 8 in the AWGN channel and the multipath Rayleigh fading channel.

**Figure 9:**
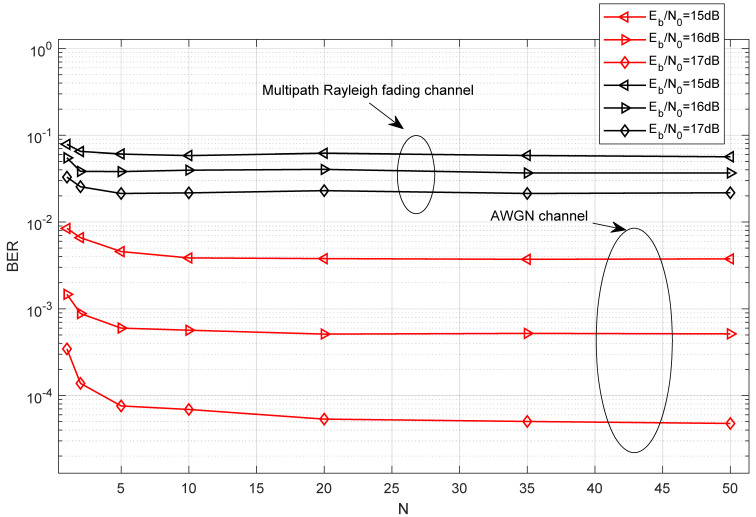
BER performance of the proposed system versus the number of the parallel groups over the AWGN channel and multipath Rayleigh fading channel with U=100,P=16,M=4,J=4.

In Figure 9, the effect of different numbers of parallel groups on the BER of the proposed system over the AWGN channel and the multipath Rayleigh fading channel is demonstrated when $\frac{E_{b}}{N_{0}}$ is fixed. The BER performance improves as the number of groups increases; however, it does not improve as the number of groups increases indefinitely. A compromise number of the parallel groups is 10.

**Figure 10:**
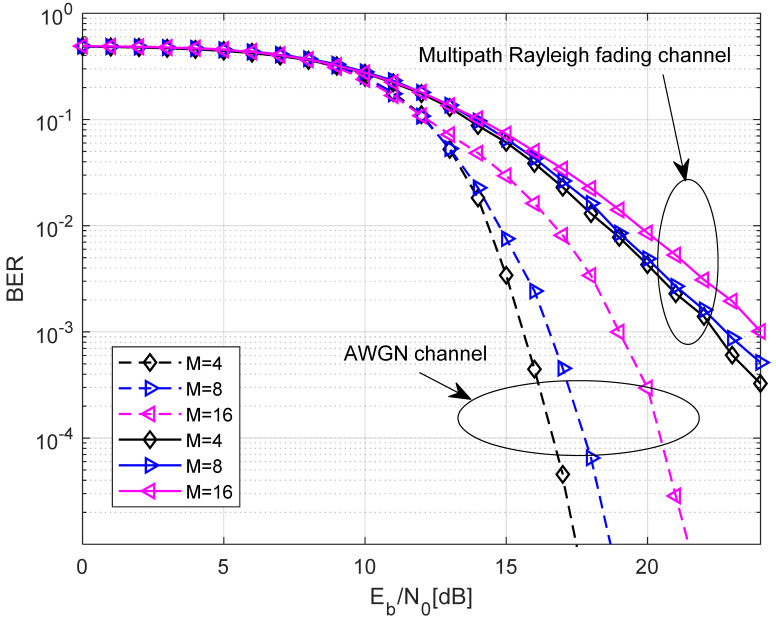
The comparison of the BER performance with different M over the AWGN channel and multipath Rayleigh fading channel with U=100,P=64,J=4,N=10.

In Figure 10, the comparison of the BER performance with different M over the AWGN channel and multipath Rayleigh fading channel with U=100,P=64,J=4,N=10 is shown. Obviously, the BER performance is best when M = 4.

**Figure 11:**
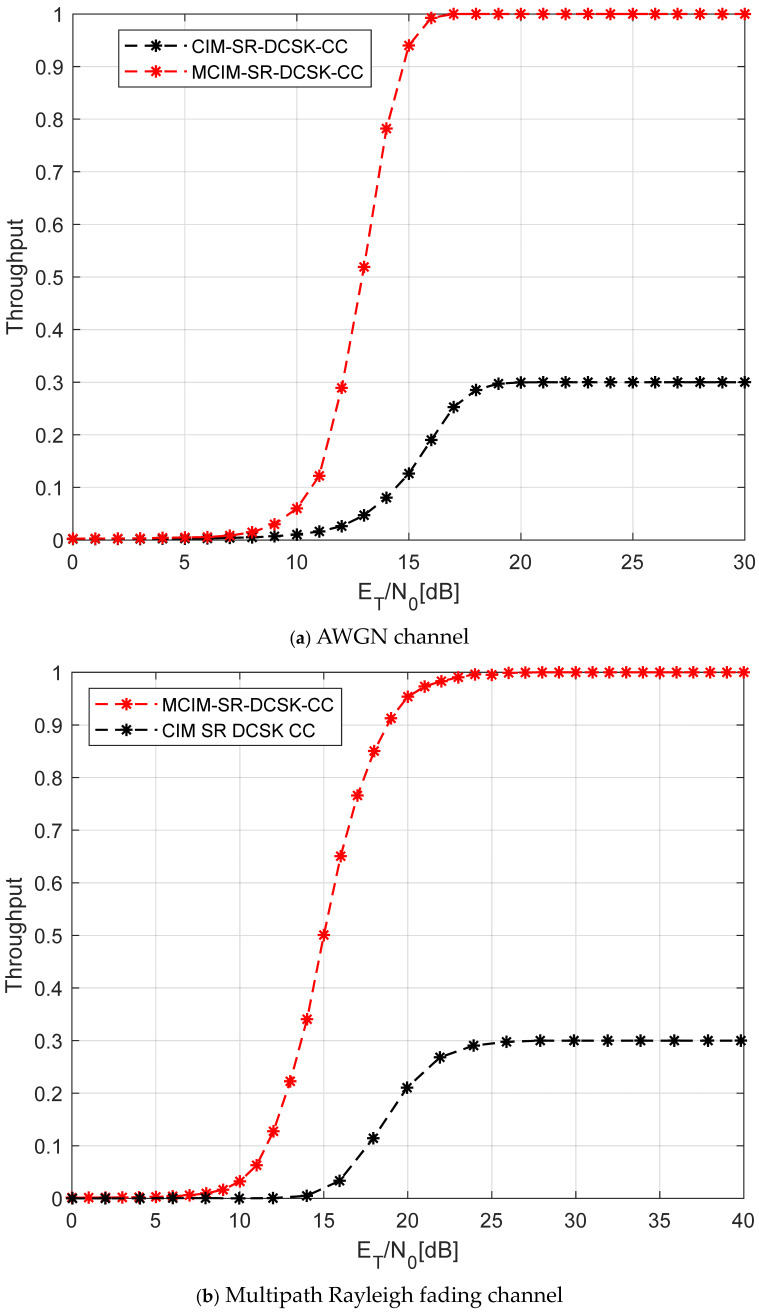
The comparison of the normalized throughputs of the proposed system.

In Figure 11, the comparison of the normalized throughputs between the proposed system and the CIM-SR-DCSK-CC system over the AWGN channel and multipath Rayleigh fading channel is shown. The normalized throughput of the proposed MCIM-SR-DCSK-CC system greatly exceeds those of the CIM-SR-DCSK-CC system. For example, at $E_{T}/N_{0}=30\;dB$, the normalized throughput of the proposed MCIM-SR-DCSK-CC system is 1 over the AWGN and multipath Rayleigh fading channels. Compared with this, those of the CIM-SR-DCSK-CC system is 0.3. This is due to the fact that the proposed system has a great improvement in time duration for information transmission with respect to the other counterpart. Here, it is assumed that $E_{T}$ represents the total transmission energy.

**Figure 12:**
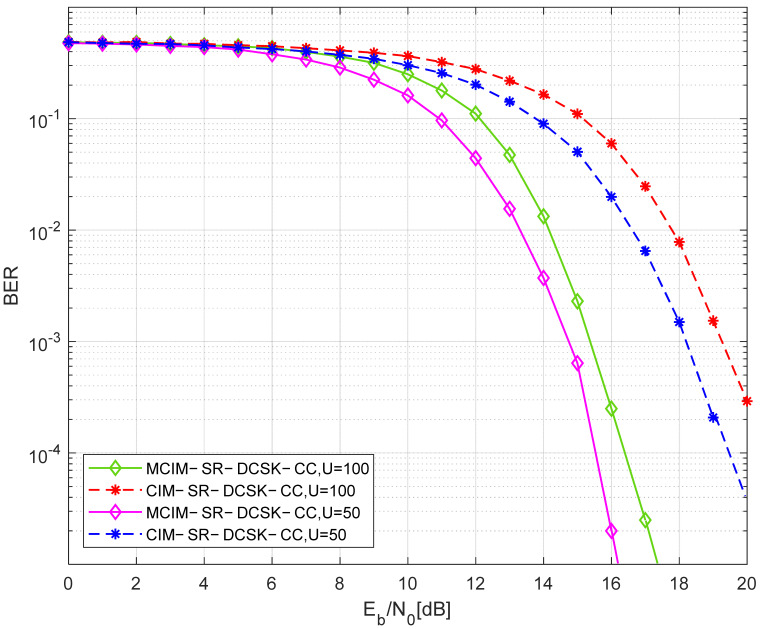
The comparison of the BER performance between the proposed system and the CIM-SR-DCSK-CC system over the AWGN channel.

In Figure 12, the comparison of the BER performance between the proposed system and the CIM-SR-DCSK-CC system over the AWGN channel is shown. The number of replications is J=4. Obviously, the BER performance of the proposed system is better than the CIM-SR-DCSK-CC system when some parameter settings are the same. For example, when U=50, the gain of the proposed MCIM-SR-DCSK-CC system is about 4 dB at a BER of $10^{-3}$ over the AWGN channel.

The proposed system is primarily targeted at high-data-rate communication systems that typically operate in moderate-to-high SNR regions. For instance, in satellite communication systems and certain urban wireless environments, the SNR often exceeds 12 dB due to the use of advanced signal processing techniques and favorable propagation conditions [39,40]. In these scenarios, our proposed system demonstrates significant performance improvements in terms of BER and throughput, making it highly relevant for applications that require high reliability and efficiency. The chosen SNR range (above 12 dB) was selected to highlight the superior performance of our proposed method in environments where high SNR is achievable. This range is particularly relevant for systems that benefit from advanced techniques such as beamforming, adaptive modulation, and error correction coding, which can effectively increase the SNR. By focusing on this range, we aim to demonstrate the potential of our method in scenarios where it can provide the most significant advantages.

## 5. Conclusions

In this paper, a new SR-DCSK cooperative communication system based on modified code index modulation has been proposed, referred to as the MCIM-SR-DCSK-CC system. In the proposed system, the relay both forwards the source information bit to the destination and employs modified code index modulation to simultaneously convey additional information bits. The throughput, EE, and the data rates of the proposed system are compared with other systems. It is shown that the proposed system has better EE, high throughput, and high data rates in comparison with its competitors. The computational complexity and the memory requirement are analyzed. The theoretical BER expressions are obtained for the proposed system over AWGN and multipath Rayleigh fading channels, which are consistent with the simulation results. According to the simulation results, it is demonstrated that the BER performance will improve when the length of the chaotic signal decreases. And the BER performance of the proposed system runs better with M=4 than M=8 and M=16. The comparison between the proposed system and the CIM-SR-DCSK-CC system demonstrates that the proposed system has better BER performance than its competitors.

## Figures and Tables

**Table 1:** Comparisons between systems.

Performance	MCIM-SR-DCSK-CC	CIM-SR-DCSK-CC
EE	$\frac{N^{2}({1+m}_{ci}+m_{cm})}{N+1}$	$\frac{N^{2}({1+m}_{ci})}{N+1}$
Data rate	$N({1+m}_{ci}+m_{cm})$	${1+m}_{ci}$

## Data Availability

Data are contained within this article.
